# Genotypic and Environmental Effects on Tocopherol Content in Almond

**DOI:** 10.3390/antiox7010006

**Published:** 2018-01-05

**Authors:** Ossama Kodad, Rafel Socias i Company, José M. Alonso

**Affiliations:** 1 Département Arboriculture-Viticulture, École Nationale d’Agriculture de Meknès, Meknès BP S/40, Morocco; osama.kodad@yahoo.es; 2 Unidad de Hortofruticutura, Centro de Investigación y Tecnología Agroalimentaria de Aragón (CITA), Av. Montañana 930, 50059 Zaragoza, Spain; jmalonsos@aragon.es

**Keywords:** almond, *Prunus amygdalus*, tocopherols, genotype, climate

## Abstract

Almond is the most important nut species worldwide and almond kernels show the highest levels of tocopherols among all nuts. In almond, tocopherols not only play a substantial role as a healthy food for human consumption, but also in protecting lipids against oxidation and, thus, lengthening the storage time of almond kernels. The main tocopherol homologues detected in almond in decreasing content and biological importance are α-, γ-, δ-, and β-tocopherol. Tocopherol concentration in almond depends on the genotype and the environment, such as the climatic conditions of the year and the growing management of the orchard. The range of variability for the different tocopherol homologues is of 335–657 mg/kg of almond oil for α-, 2–50 for γ-, and 0.1–22 for β-tocopherol. Drought and heat have been the most important stresses affecting tocopherol content in almond, with increased levels at higher temperatures and in water deficit conditions. The right cultivar and the most appropriate growing conditions may be selected to obtain crops with effective kernel storage and for the most beneficial effects of almond consumption for human nutrition and health.

## 1. Introduction

Almond is the most important tree nut crop in terms of commercial production [1]. An adaptation to harsh climates combined with an ability to develop a deep and extensive root system has allowed almond to exploit a wide range of ecological niches. Almond is well-adapted to the Mediterranean climate, characterized by mild winters and dry, hot summers. This adaptation has led to early bloom and rapid early shoot growth because of the low chilling requirements of almond. Almond also shows high tolerance to summer drought and heat. Almond has traditionally been the earliest temperate fruit tree crop to bloom, which limited growing to areas relatively free from spring frosts before the release of late-blooming cultivars by different breeding programs, since frosts at bloom or early fruit development can reduce, and even completely nullify, the crop. Since almond is naturally self-incompatible, it often requires cross-pollination, which further acts to promote genetic variability and adaptability to diverse environments [2].

The edible part of the almond nut is the kernel, considered an important food crop with a high nutritional and medicinal value. The oldest and most extensive medical system that first recorded the health uses for almonds derives from ancient Greeks and then the Persians, and later in traditional Chinese medicine and Indian Ayurvedic medicine [3]. From medieval times to the 18th century, almond nuts were a source of substitute “milk” [4], and also it was used as thickener before starch was “discovered” [3]. Almond consumption has almost doubled in the last 20 years [5], a fact that highlights how this consumption has evolved from a convenient snack food and component of a high number of confectioneries, to an important food which is increasingly recognized as essential for maintaining and increasing human health. Recent nutritional and medical studies have associated the regular consumption of almonds with a wide range of health benefits, including protection from cancer [6,7], obesity [8,9,10], diabetes [11,12], and heart diseases [13,14,15,16,17]. Almond kernels may be consumed in many different ways, blanched or unblanched, raw or combined, and/or mixed with other nuts. They can also be transformed to produce marzipan, nougat, almond milk, and almond flour, incorporated in many pastries and ice creams. Almonds may also be combined in other products and gastronomic specialties [18]. The high nutritive value of almond kernels is mainly due to their high lipid content. This lipid fraction, even constituting an important source of calories, does not contribute to cholesterol formation because of their high level of unsaturated fatty acids, mainly mono-unsaturated fatty acids [19]. Although almond kernels are high in energy, humans compensate their effect with their high satiety value [20]. The absorption of energy from almond kernels is rather inefficient, having been suggested that their chronic consumption may raise resting energy expenditure [21]. Acute and longer-term almond ingestion may help in regulating body weight [17], modulating fluctuations of blood glucose [22], total low density/high density lipoprotein cholesterol ratio, and triglycerides [23].

Almond kernel quality must be high in order to fulfill not only the industry requirements, but also to be attractive for the consumers [24]. Until recently only the kernel physical traits were considered when trying to establish almond quality [18], but the kernel chemical composition appears to be essential when establishing the best raw material for the different industrial applications and the high diversity of almond confectioneries [24]. In view of the high lipid fraction in the almond kernel, the quality of the almond oil is considered as the most important feature in the evaluation of almond quality. Different parameters related to the lipid fraction have been suggested for quality evaluation of the almond kernels, such as the amount of oil content in the kernel (fat percentage over the kernel dry weight), the percentage of oleic acid of the total fatty acids, the ratio of the percentages of oleic/linoleic acids (O/L), and, especially, the tocopherol concentration in the almond oil [24,25].

Early reviews on the composition of the almond nuts [18,26] did not include tocopherols as an important component of almond kernels. More recently tocopherols were already included in several reviews [27,28], but most of them were primarily descriptive without attempts to assess quality, particularly as it relates not only to industrial requirements, but also to breeding goals and approaches. Extensive variability in the chemical composition has been demonstrated among cultivars; additionally the importance of differences in geographical origins, as well as climatic and growing conditions have also been demonstrated. Despite this extensive information, little is known concerning the genetic control and inheritance of biochemical components of almond quality. For all these reasons, this review summarizes the current knowledge of the almond kernel tocopherol composition and factors affecting its variability.

## 2. Tocopherol Variability in Almond

Almost all studies carried out on vitamin content in almond are limited to those having an antioxidant effect, mainly tocopherols. The main tocopherol homologues detected in almond in decreasing importance are α-, γ-, δ-, and β-tocopherol. The main biochemical function of tocopherols is considered to be the protection of polyunsaturated fatty acids against peroxidation [29]. They also have protective roles in human health since recent data indicate that they have hypo-cholesterolemic, anti-cancer, and neuroprotective properties [30]. Tocopherol concentrations have been determined in many vegetable oils, having been correlated with their antioxidant activity, since this activity may depend on the ratio of % total tocopherols/% polyunsaturated fatty acids [31]. Tocopherol concentration plays an important role in protecting almond lipids against oxidation, thus increasing the possibilities of lengthening kernel storage [31,32,33].

The range of variability of the different tocopherol homologues has been reported for different almond cultivars and genotypes from different countries [27,34,35,36,37,38,39,40,41,42,43,44], being summarized in Table 1. The most biologically active form of vitamin E is α-tocopherol, being preferentially utilized in the human body over the other homologues [45], ranging from 200 mg/kg oil in some Australian cultivars [44] to 656.7 mg/kg oil in some local accessions from Morocco [37]. At high temperatures γ-tocopherol has been reported to be much more effective as an inhibitor of polymerization and protection against oxidation than α-tocopherol [46,47]. The range of variability of this homologue was from 2.4 mg/kg oil in some local Moroccan almond seedlings [37] to 50.2 mg/kg oil in some almond selections from the CITA breeding program [35,48]. For δ-tocopherol, the range of variability is reduced as compared to the other homologues and ranged from 0.1 mg/kg oil in some local Moroccan almond seedlings [37] to 22.0 mg/kg oil in some Spanish almond cultivars [39].

## 3. Environmental Effects

Tocopherol levels increase in response to a variety of abiotic stresses, considered as evidence of its protective role [49]. Tocopherol concentration in almond oil depends on the genotype and the climatic conditions of the year [36,38,39,40,42], as well as on the environmental conditions of the growing region [36,38]. Drought and heat have been the most important stresses studied until now on the expression of the chemical compounds in fruit trees, including almond. The climatic conditions of the year, mainly temperature, affect the concentration of the different tocopherol homologues in several nut crops [50], indicating that these components depend on the temperature and the occurrence of drought during fruit or nut growth.

The effect of drought stress on the oil percentage in almond kernels and on its composition is not clear since the results of different studies undertaken in this field are ambiguous. However, studies in other species reported that water stress appears to promote tocopherol synthesis [51,52,53]. In almond there was no obvious relationship between almond tocopherol content and the degree of water deficiency [54], since little variation in kernel oil tocopherols was observed under moderate water stress, except for the more severe deficit of 70% SDI (sustained deficit irrigation) which for all components, except γ-tocopherol, had higher values than the control. In this study, the minor tocopherol homologues appeared to be slightly more responsive to deficit irrigation than the main homologue, α-tocopherol, but their small proportion had little impact on the amount of the total tocopherol content [54]. In the “Nonpareil” almond cultivar, the highest accumulation rate of α-tocopherol takes place during the period from 74 to 94 days after anthesis [44], indicating that the critical period to add nutrient and water to ensure normal accumulation of these chemical compounds is during this period. Tocopherol concentration has been reported to be affected by drought stress in olive oil [55], but not in *Brassica napus* [56], by temperature in soybean [57], and by a combination of both in shea butter (*Vitellaria paradoxa* C.F. Gaertn.) [50]. In olive oil, the total tocopherols and α-tocopherol were highly influenced by the crop-year rainfalls, with the highest concentration from olives harvested during the driest year [58]. The increase in tocopherol content might contribute to the prevention of plant oxidative damage in drought conditions [59,60].

Higher tocopherol concentrations were found in almond kernels harvested in years with higher temperatures suggesting that this environmental factor could affect tocopherol synthesis during kernel development [34]. A study on the effect of two contrasting environments, with drought and heat conditions in Morocco, and irrigated and cold conditions in Spain, concluded that the tocopherol content, mainly of α-tocopherol, increased under the warmer climate conditions of Morocco [37]. More recently, higher α-tocopherol concentrations (~646 µg/g in average) were found when the almond kernel development mostly coincided with spring and summer months with warmer mean temperature in a study in arid Northwestern Argentina [40]. Similar results were reported for α-tocopherol in some almond genotypes grown under hot and dry conditions in Afghanistan [61]. Temperature during seed development in sunflower has shown to affect oil yield and tocopherol concentration since high temperatures may have a negative effect in oil synthesis, but not in tocopherol concentration [62,63]. In soybean seed, it has been reported that the increasing temperature from 23 °C to 28 °C can significantly increase tocopherol levels [64]. Thus, the temperature could be considered an important parameter playing a great role in tocopherol accumulation in different species, including almond.

Concerning the effect of solar radiation, in “Nonpareil” almond kernels the concentration of α-tocopherol was increased after a mild solar UV radiation supplement using the white weed mat, which may have altered metabolic pathways and stimulated α-tocopherol accumulation in almond lipids [44]. In sunflower, it has been reported that an increase in intercepted solar radiation per plant increased the amount of tocopherol per grain [65].

## 4. Genetic Effects

The evaluation of tocopherol concentration in the oil of different almond cultivars and genotypes showed high variability of the different tocopherol homologues, with a great effect of the environment [28]. However, it has been reported that the cultivars “Atocha”, “Desmayo Rojo”, “Desmayo Largueta” from Spain, and “Ferraduel” from France showed stable and similar year to year values for α-tocopherol; whereas “Atocha”, “Ferraduel”, and “Fournat de Brézenaud”, and “Yaltinskij” from Ukraine were also stable for γ-tocopherol content in almond kernels produced under contrasting climatic conditions, with drought and heat conditions in Morocco, and irrigated and cold conditions in Spain [36]. These results confirmed that the stability of each tocopherol homologue depends on the specific characteristics of the genotype [34,35]. The estimation of the heritability of the different tocopherols isomers were estimated for the first time in almond [35], reporting that the content of γ-tocopherol showed high heritability estimates, with h^2^ = 60.0%, whereas α-tocopherol showed lower heritability (h^2^ = 20.5%). These results confirm that the tocopherol content in almond oil kernel is under polygenic control, as previously suggested [28].

The biosynthetic pathway of vitamin E in plants was biochemically elucidated several years ago, and all enzymes in this pathway were localized to the inner chloroplast envelope [66,67,68]. Today, the availability of complete genome sequences, in particular from *Arabidopsis* and *Synechocystis* sp. PCC6803, all biosynthetic genes in tocopherol biosynthesis have been identified and cloned to date [69,70]. A mutation in the gene VTE5 (PCT) of *Arabidopsis* lead to the discovery of its function, since it is encoding a protein with phytol kinase activity, directly involved in the biosynthetic pathway of tocopherol [70,71]. Tocopherol QTL analysis found that up to 65% of the markers were co-located in certain genomic regions of maize, including the candidate genes PDS1 (*p*-hydroxyphenylpyruvate dioxygenase; HPPD) and VTE4 (γ-tocopherol methyltransferase; γ-TMT), thus showing that a single QTL may affect more than one tocopherol homologue [71]. After exploring mutant inbred lines in sunflower, three loci (*m = Tph*1, *g = Tph*2, and *d*) were shown to disrupt synthesis in α-tocopherol production; additionally, the loci losing function in these mutations enhanced synthesis of other tocopherols [72]. In almond no studies have been conducted to elucidate the pathway biosynthesis of tocopherol isomers and the genes involved in this process, but, recently, five different QTLs believed to control the tocopherol concentration in the almond kernel oil have been identified [73]. More studies are required to understand the biosynthetic pathway of these biochemicals components to elucidate the gene involved in these pathways. This information will be of great interest for the breeders to improve tocopherol content in the future, since the almond kernel is used more and more in different industrial processes using high temperatures in their application [24].

## 5. Conclusions

The present information on the different effects on tocopherol content in almond is scarce. Only descriptive results have been published, not allowing a critical comparison among them, since some results are given as tocopherol content in the almond kernel and others in the kernel oil, not always stating the oil concentration of the almond kernel. Although genetic and environmental effects affecting tocopherol content in almond have been described, no interaction between them has ever been established. The present scenario of climatic change and of the shift of almond growing to warmer regions [1] may have a positive effect on the tocopherol content in almond kernels due to the effect of higher temperatures increasing this content. Consequently, the right cultivar and the most appropriate growing conditions may be selected in order to have almond crops not only allowing a better effective kernel storage, but also holding the most beneficial effects for human nutrition and health with their consumption. The objective of selecting for high tocopherol content in a breeding progeny is easily attainable because of its high heritability, whenever the adequate parents are chosen. As a consequence, these components could be considered as selection criteria in almond breeding programs as already suggested [24]. At present the possibilities of selecting cultivars, growing regions, and orchard practices offer an optimistic outlook for increasing tocopherol content in almond.

## Figures and Tables

**Table 1:** Tocopherol homologue concentration in almond kernel and kernel oil.

Tocopherol Homologue	Range of Variability		Origin	Reference
	mg/kg Kernel	mg/kg Oil		
α-tocopherol	85–190		Spain	[42]
		335.3–551.7		[39]
		309–656.7	Morocco	[37]
	180–320		California	[38]
	250–840		Italy	[32]
		350–471		[43]
		370–571	Argentina	[40]
		200	Australia	[44]
β-tocopherol	50–80		Italy	[32]
	1.2		Australia	[44]
γ-tocopherol		6.1–50.2	Spain	[34,39]
	1.4–8.4			[42]
		75	Italy	[31]
		2.4–13.5	Morocco	[37]
	5.7		Australia	[44]
δ-tocopherol	0.2–1.6		Spain	[42]
		0.2–22		[34,39]
		0.1–0.3	Morocco	[37]
